# Association Between Online Health Information–Seeking Behaviors by Caregivers and Delays in Pediatric Cancer: Mixed Methods Study in China

**DOI:** 10.2196/46953

**Published:** 2023-08-16

**Authors:** Jiamin Wang, Xuemei Zhen, Peter C Coyte, Di Shao, Ni Zhao, Lele Chang, Yujia Feng, Xiaojie Sun

**Affiliations:** 1 Centre for Health Management and Policy Research School of Public Health, Cheeloo College of Medicine Shandong University Jinan China; 2 National Health Commission Key Lab of Health Economics and Policy Research Shandong University West-Wenhua Road, 44 Jinan China; 3 Institute of Health Policy, Management and Evaluation Dalla Lana School of Public Health University of Toronto Toronto, ON Canada

**Keywords:** online health information–seeking behaviors, patient delay, diagnostic delay, treatment delay, mixed methods study

## Abstract

**Background:**

Pediatric cancer patients in China often present at an advanced stage of disease resulting in lower survival and poorer health outcomes. One factor hypothesized to contribute to delays in pediatric cancer has been the online health information–seeking (OHIS) behaviors by caregivers.

**Objective:**

This study aims to examine the association between OHIS behaviors by caregivers and delays for Chinese pediatric cancer patients using a mixed methods approach.

**Methods:**

This study used a mixed methods approach, specifically a sequential explanatory design. OHIS behavior by the caregiver was defined as the way caregivers access information relevant to their children’s health via the Internet. Delays in pediatric cancer were defined as any one of the following 3 types of delay: patient delay, diagnosis delay, or treatment delay. The quantitative analysis methods included descriptive analyses, Student t tests, Pearson chi-square test, and binary logistic regression analysis, all performed using Stata. The qualitative analysis methods included conceptual content analysis and the Colaizzi method.

**Results:**

A total of 303 pediatric cancer patient-caregiver dyads was included in the quantitative survey, and 29 caregivers completed the qualitative interview. Quantitative analysis results revealed that nearly one-half (151/303, 49.8%) of patients experienced delays in pediatric cancer, and the primary type of delay was diagnosis delay (113/303, 37.3%), followed by patient delay (50/303, 16.5%) and treatment delay (24/303, 7.9%). In this study, 232 of the 303 (76.6%) caregiver participants demonstrated OHIS behaviors. When those engaged in OHIS behaviors were compared with their counterparts, the likelihood of patient delay more than doubled (odds ratio=2.21; 95% CI 1.03-4.75). Qualitative analysis results showed that caregivers’ OHIS behaviors impacted the cancer care pathway by influencing caregivers’ symptom appraisal before the first medical contact and caregivers’ acceptance of health care providers’ diagnostic and treatment decisions.

**Conclusions:**

Our ﬁndings suggest that OHIS among Chinese pediatric caregivers may be a risk factor for increasing the likelihood of patient delay. Our government and society should make a concerted effort to regulate online health information and improve its quality. Specialized freemium consultations provided by health care providers via online health informatic platforms are needed to shorten the time for caregivers’ cancer symptom appraisal before the first medical contact.

## Introduction

Detecting pediatric cancer at an early stage is one of the effective means of improving survival rates and reducing treatment toxicity rates. This is particularly crucial for pediatric cancer patients who are in the early stage of their life course [1,2]. Pediatric cancer is the second leading cause of death among children aged younger than 14 years worldwide [3,4]. With the significantly higher incidence rates and relatively lower survival rates of pediatric cancer, great gaps for pediatric cancer treatment in China still exist [5]. China faces the issue of an advanced stage of pediatric cancer at presentation, and a significant proportion of patients delay seeking help after the self-discovery of symptoms of pediatric cancer. Delays in pediatric cancer care may influence the effectiveness of and patient’s experience with cancer care, with impacts on the subsequent prognosis.

**Figure 1:**
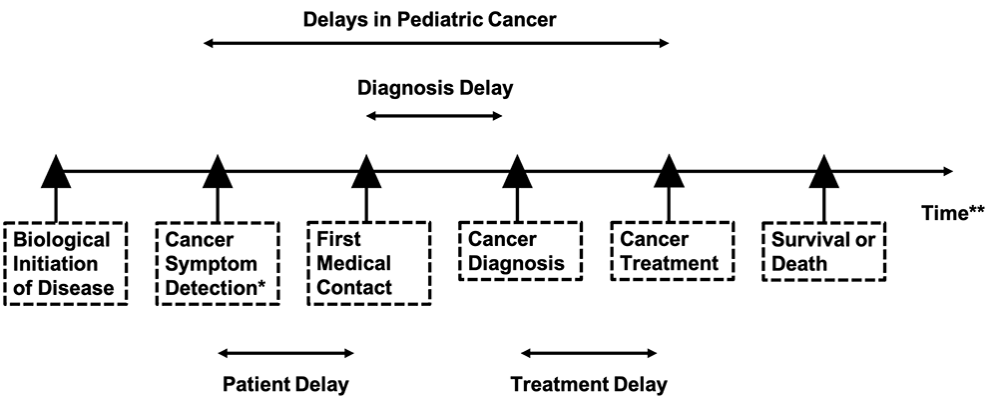
Definition of delay variables in the cancer care pathway. *Cancer symptom detection defined by date of first parental-reported symptom, **Similar distances between time points are not representative of actual time intervals.

The definitions of delays in pediatric cancer vary between different studies. In early studies, researchers preferred to adopt broad concepts of delays. Robbin and colleagues [6] defined delays as intervals between the onset of symptoms and treatment, then the concept of delays was divided specifically by different time cutoffs. Tam et al [7] characterized different components of delays from biological initiation of disease to the end of the life for Canadian pediatric cancer patients (Figure 1). The same division was widely used for pediatric cancer both in high-income [8,9] and low-income countries [10]. In this study, a patient delay was defined as ≥30 days between the first onset of symptoms and the first medical consultation [11,12], diagnosis delay was defined as a period ≥2 weeks between the first medical visit for the symptoms and diagnosis of cancer [13], and treatment delay was defined as ≥14 days between the date of the onset of symptoms and the start of treatment [14].

Caregivers’ online health information–seeking (OHIS) behavior has an ambiguous effect on delays in pediatric cancer. Caregivers’ OHIS behavior is defined as the way caregivers access information relevant to their children’s health via the Internet, such as websites, online support groups, forums, and social media [15-17]. Misinformation and fake news on the Internet might prolong delays for pediatric cancer patients. Sajid and colleagues [18] showed that online health information for cancer patients is commercialized and needs to be regulated to improve the quality. Ng et al [19] demonstrated that one of the most common knowledge barriers resulting in delay is a lack of knowledge and misinformation from the Internet or other social media platforms. However, a qualitative study from the perspective of pediatric cancer caregivers in Denmark demonstrated that OHIS behaviors by caregivers could be useful to shorten delays for patients with pediatric cancer by speeding up contact with health care systems [20,21]. Therefore, the relationship between caregivers’ OHIS behaviors and delays in pediatric cancer is still unclear.

There are other associated factors for delays for pediatric cancer patients in addition to caregivers’ OHIS behaviors. Previous studies have shown that patient age [1,9,22-26], gender [27], and cancer type [9,25]; caregiver economic status [23]; access to health care facilities [28]; caregiver educational level; and first consulted health professional [26,29,30] are influencing factors for delays for pediatric cancer patients. In addition, Dang-Tan and Franco [31] grouped these factors into 3 categories: patient or parent, disease, and health care. Haimi and colleagues [32] had similar opinions that divided associated factors into health care system–related parameters, patient-related parameters, and cancer-related parameters. These associated factors should be considered when analyzing the relationship between caregivers’ OHIS behavior and delays in pediatric cancer.

Few studies have investigated the association between caregivers’ OHIS behavior and delays in pediatric cancer care, and the results remain inconsistent. By addressing this gap, the growing literature will be further extended. In summary, we had 2 overarching research objectives: (1) to examine the association between caregivers’ OHIS behavior and delays in Chinese pediatric cancer using quantitative analysis and (2) to explore the role caregiver’ OHIS behavior plays on the cancer care pathway using qualitative analysis.

## Methods

### Conceptual Framework

**Figure 2:**
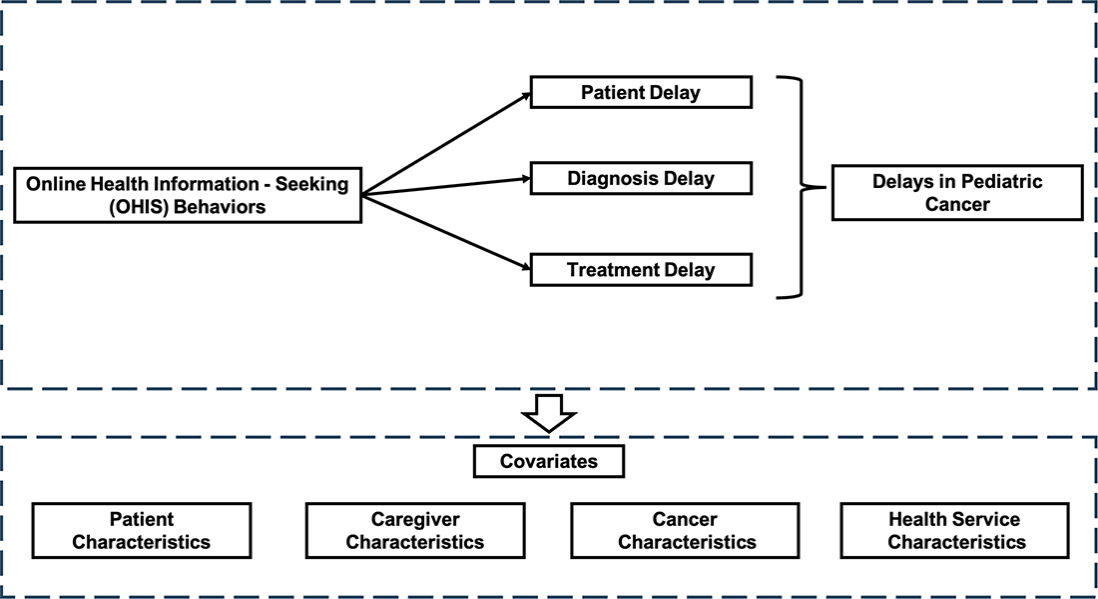
Hypothesized model.

Figure 2 illustrates the guided conceptual framework in this study. Causation is hypothesized to flow from the OHIS behaviors by caregivers to the delays for Chinese pediatric cancer patients, with an expected positive correlation between these constructs. Delays for pediatric cancer patients are comprised of 3 parts: patient delay, diagnosis delay, and treatment delay. The positive correlation between OHIS behaviors by caregivers and delays in pediatric cancer is potentially confounded by covariates such as patient, caregiver, cancer, and health service characteristics. Previous literature has explored this formulation [18,19,33], and we assess these associations in the Chinese context in this study.

### Study Design

In this study, we used mixed methods, specifically the sequential explanatory approach [34], which involves 2 phases: a quantitative analysis first, followed by an informed qualitative analysis phase [29,30,34]. In this study, quantitative methods were first used to examine the association between caregivers’ OHIS behaviors and the risk of delays among pediatric cancer patients. A cross-sectional study using self-reported questionnaires was conducted in this period. The quantitative results informed the sampling of the following qualitative study, which allowed us to connect the quantitative component of the study to the qualitative component [34]. Qualitative interviews were semistructured with open-ended questions and standardized prompts. Qualitative methods were then used to explore the impact caregivers’ OHIS behaviors had on the cancer care pathway among Chinese patients with pediatric cancer. After the completion of qualitative analysis, we performed the second stage of data integration, during which we used the qualitative findings to explain, enrich, justify, or challenge the quantitative results [29,30,34,35]. The entire study was organized by conducting the quantitative analysis first, followed by the qualitative analysis.

### Study Sampling

**Figure 3:**
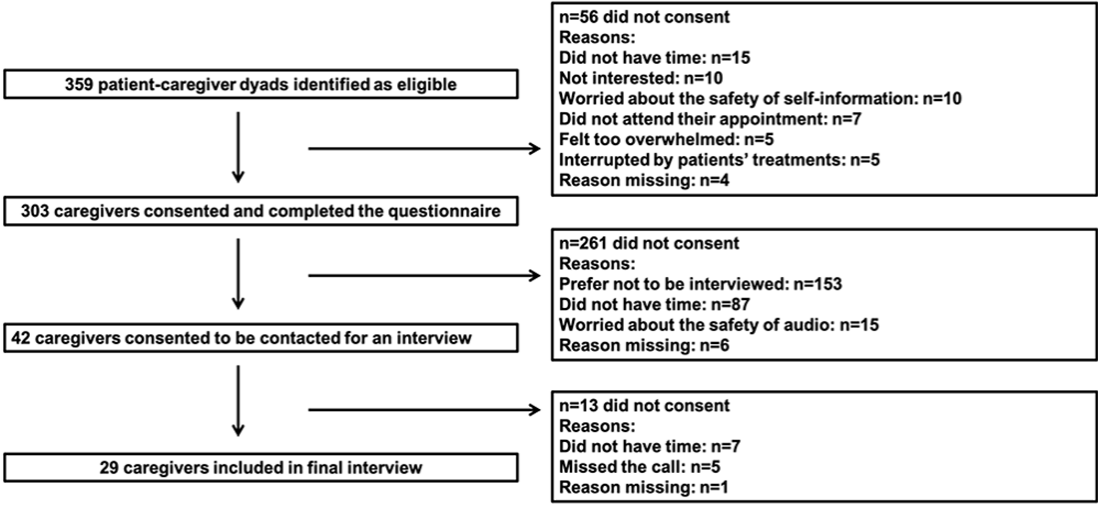
Participant recruitment flow diagram.

Cluster sampling was conducted in 3 regional hospitals for pediatric oncology treatment in Jinan, Shandong province, China, from May 2021 to August 2021. Shandong province is an important coastal province with over 100 million people in East China. The largest specialized tumor hospital in the province, best general hospital in the province, and only specialized pediatric hospital in the provincial capital comprised the study setting. The 3 hospitals have leading capabilities in pediatric blood tumors or solid tumors and are representative pediatric tumor treatment institutions in Shandong province. This study recruited 149 patients with solid tumors from the specialized tumor hospital, 101 patients with hematological tumors from the general hospital, and, finally, 53 patients with hematological tumors from the specialized pediatric hospital. Although 359 potential patient-caregiver dyads were invited to participate, the sample of respondents was composed of 303 patient-caregiver dyads who consented and were able to complete all data requirements, thereby yielding a response rate of 84.4%. A study flowchart of participant selection is shown in Figure 3. Included pediatric cancer patients were aged under 14 years old, required a malignant diagnosis, and were being treated during the study period in the aforementioned 3 hospitals. The family caregivers who refused to participate and those who consented but were unable to participate due to a serious developmental, audiovisual, or cognitive impairment were excluded. Data were collected through face-to-face interviews by trained postgraduates.

Purposive sampling [34,36] of the following qualitative study was informed by the quantitative results. There were 42 caregivers who consented to be contacted for an interview, and 29 caregivers were involved in the final interview. More detailed information is found in Figure 3. Caregivers engaged in OHIS behaviors between the onset of symptoms and the start of treatment and those whose children had delays in their pediatric cancer were included. Eligible caregiver respondents were interviewed by 2 well-trained postgraduate students, who were trained for >7 days to master the survey skills and be familiar with the content. Interviews were conducted at sample hospitals. Before the interview, all participants received a consent form that outlined the possible risks and benefits of participating, the commitment to protecting all research data, the permission for recording, and the ability to opt out of the survey at any time. The interview outline was based on previous literature reviews.

### Study Measures

#### Delays in Pediatric Cancer

Previous literature characterized different components of delays as patient delay, diagnosis delay, and treatment delay (Figure 1) [13,37]. Information on patient delay was collected via a question asking, “What were the time intervals between you noticing the symptoms and your first medical visit?” If the time interval was ≥30 days, the patient was classified as experiencing a patient delay. Information on diagnosis delay was collected via a question asking, “What were the time intervals between your first medical visit and the diagnosis of pediatric cancer?” If the time interval was ≥14 days, the patient was classified as having a diagnosis delay. Information on treatment delay was collected via a question asking, “What were the time intervals between the diagnosis of pediatric cancer and the start of treatment?” If the time interval was ≥14 days, the patient was classified as having a treatment delay. Finally, for the delays in pediatric cancer, if the patients had any one of the 3 types of delay (patient delay, diagnosis delay, or treatment delay), he or she was classified as having delays in pediatric cancer.

#### Caregivers’ OHIS Behavior

OHIS behavior by the caregiver was defined as the way caregivers access information relevant to their children’s health via the Internet, such as websites, online support groups, forums, and social media [15-17]. Information on caregivers’ OHIS behaviors in this study was obtained via a question asking, “Did you engage in OHIS between the onset of symptoms and the start of treatment? (a) Yes, (b) No.”

#### Covariates

Based on previous literature reviews [9,32], we adopted the commonly used classification of associated factors for delays in pediatric cancer, which included patient, caregiver, cancer, and health service characteristics [31,32], with each set of characteristics derived from the study questionnaire. Patients’ characteristics consisted of age, gender (male or female), and private insurance coverage (yes or no). Caregivers’ characteristics included age, gender (male or female), educational attainment (junior high school or below, high school or above), marital status (married or not), residency (urban or rural), occupation (employed or unemployed), private insurance (yes or no), number of children (1 or ≥2), household income (≤¥30,000 or >¥30,000 per year), and family cancer history (yes or no). Cancer characteristics comprised cancer type (pediatric leukemia or pediatric solid tumors). Health service characteristics included the distance to a primary care provider (≤5 km or >5 km), distance to pediatric cancer specialists in Shandong province (within Shandong province or out of Shandong province), and the number of health care providers (HCPs) visited before the diagnosis (1 or >1).

### Qualitative Study

Qualitative interviews were semistructured with open-ended questions and standardized prompts. The interview topic guide covered caregivers’ OHIS behavior and the help-seeking process from noticing cancer symptoms to receiving cancer treatment. Interviews were conducted until saturation was achieved.

### Study Analysis

For the quantitative analysis, descriptive analyses were used to describe the sociodemographic characteristics of pediatric cancer patients and caregivers. Continuous variables were summarized as mean (SD). Student *t* tests were used to examine the difference in means for continuous variables among participants with delays and those without. Categorical variables are presented as the proportion (%) and were compared using the Pearson chi-square test. Binary logistic regression analyses were performed to estimate odds ratios (ORs) and 95% CIs for the association between the caregiver’s OHIS behavior and the risk of delays in pediatric cancer. Caregivers without OHIS behaviors were used as the reference group. The unadjusted model examined the association between caregivers’ OHIS behaviors and delays in pediatric cancer care without adjustment for any covariates. Model 1 included the patients’ characteristics (age, gender, and private insurance). Model 2 made an additional adjustment for the caregivers’ characteristics (age, gender, education level, marital status, residency, occupation, private insurance, number of children, household income, and family cancer history). Finally, Model 3, the fully adjusted model, included the covariates in Model 2 plus cancer characteristics (cancer type) and health care service characteristics (distance to a primary care provider, distance to pediatric cancer specialists in Shandong province, and the number of HCPs visited before diagnosis). These covariates have previously been reported to be associated with the risk of delays in pediatric cancer. The statistical analyses were performed using Stata 15.0 (Stata Corp).

For the qualitative analysis, all interviews were transcribed verbatim by researchers and double-checked against the recording for accuracy. To protect participants’ confidentiality, participants were given pseudonyms, and transcripts were deidentified. ﻿The first interview transcripts were reviewed by 2 researchers to reduce subjective errors, and an initial round of coding was conducted using conceptual content analysis [38] and the Colaizzi method [39,40]. The analysis process involved the following 7 stages [39-41]: (1) Each transcript was read several times by 2 researchers to ensure familiarity and make sense of the content in its entirety. (2) Each caregiver’s transcript was critically re-read by 2 researchers. The phrases and sentences directly related to the research objective were highlighted by both researchers. The 2 researchers then compared their work and came to a consensus. (3) Formulated meanings from significant statements were coded into different categories. (4) Categories were grouped into clusters of themes. Thematic clusters related to a particular issue comprised an emerging theme. (5) An exhaustive description for each of the themes was developed. The findings were reviewed by a third, senior researcher with expertise in this field of study, to confirm the accuracy of the descriptions. (6) After review by the third researcher, relevant descriptions were further modified for clarity. (7) Researchers then shared the fundamental structure statement with participants to confirm whether it accurately captured their experiences. Coding and analysis were performed using QSR NVivo12.

### Ethical Considerations

Medical ethics approval of this study was obtained from the Ethical Committee of School of Public Health, Shandong University (No. 20210403). Before distributing the questionnaires and conducting the interviews, all participants received an explanation about the nature, purpose, duration, and voluntariness of the study, and they were asked to sign consent forms. The study was conducted in accordance with the Declaration of Helsinki. Each patient and caregiver are identified as a sequential number, and only the main researcher could link the code to the identity.

## Results

### Association Between Caregivers’ OHIS Behaviors and Delays in Pediatric Cancer

Of the 303 participants, there were more patients (197/303, 65%) whose caregivers reported OHIS behaviors than those who did not. For patient characteristics, most patients were male (179/303, 59.1%) and the mean age was 6.24 (SD 0.20) years. For caregiver characteristics, younger caregivers (*P*=.02) and caregivers with more than 1 child (*P*=.03) were more likely to have delays in pediatric cancer. A large proportion of caregivers was married (287/303, 94.7%) and without a family history of cancer (274/303, 90.4%), and the overwhelming majority (253/303, 83.5%) had private insurance. For cancer and health service characteristics, patients with pediatric solid tumors (*P*<.001) had significantly more delays compared with patients with pediatric leukemia, and patients living far away from pediatric cancer specialists (*P*<.001) also had higher possibilities of delays. More detailed information is presented in Table 1.

**Table 1:** Summary characteristics of pediatric cancer patient-caregiver dyads in China, 2021 (n=303).

Characteristics			Total sample		Delay in pediatric cancer				*P* value
				Yes (n=151)		No (n=152)			
Online health information seeking (Yes), n (%)			197 (65)		106 (70.2)		91 (59.9)		.059
**Patient characteristics**									
	Age (years), mean (SD)	6.24 (0.20)		6.14 (3.38)		6.34 (3.56)		.61	
	Gender (male), n (%)	179 (59.1)		89 (58.9)		90 (59.2)		.96	
	Private insurance (No), n (%)	253 (83.5)		129 (85.4)		124 (81.6)		.37	
**Caregiver characteristics**									
	Age (years), mean (SD)	35.74 (0.34)		34.91 (5.73)		36.56 (6.11)		.02	
	Gender (female), n (%)	221 (72.9)		112 (74.2)		104 (68.4)		.27	
	Educational status (high school and higher), n (%)	152 (50.2)		76 (50.3)		76 (50)		.95	
	Number of children (≥2), n (%)	220 (72.6)		101 (66.9)		119 (78.3)		.03	
	Married (yes), n (%)	287 (94.7)		141 (93.4)		146 (96.1)		.30	
	Occupation (unemployed), n (%)	169 (55.8)		83 (55)		86 (56.6)		.78	
	Private insurance (no), n (%)	276 (91.1)		138 (91.4)		138 (90.8)		.85	
	Residency (urban), n (%)	161 (53.1)		85 (56.3)		76 (50)		.27	
	Family cancer history (no), n (%)	274 (90.4)		136 (90.1)		138 (90.8)		.83	
	Household income per year (>¥30,000), n (%)	185 (61.1)		92 (60.7)		93 (61.2)		.69	
**Cancer characteristics**									
	Cancer type (pediatric leukemia)	153 (50.5)		43 (28.5)		110 (72.4)		<.001	
**Health service characteristics**									
	Distance to a primary care provider (>5 km), n (%)	179 (59.1)		85 (56.3)		94 (61.8)		.33	
	Distance to a pediatric cancer specialist in Shandong province (out of Shandong province), n (%)	208 (68.7)		89 (58.9)		119 (78.3)		<.001	
	Number of health care providers visited before diagnosis (1), n (%)	203 (67)		91 (60.3)		112 (73.7)		.01	

Approximately one-half (151/303, 49.8%) of the patients experienced delays before treatment; the types of delays were diagnosis delay for 37.3% (113/303), followed by patient delay for 16.5% (50/303) and treatment delay for 7.9% (24/303). Table S1 in Multimedia Appendix 1 shows the correlation matrix for the 5 key study variables. Caregivers’ OHIS behavior (*r*=0.1210, *P*<.05) was only positively related with a patient delay in pediatric cancer. Table 2 shows the unadjusted and multivariate-adjusted ORs and 95% CIs for patient delay according to whether caregivers of patients with pediatric cancer had OHIS behaviors. Compared with those whose caregivers did not report OHIS behaviors, patients whose caregivers reported OHIS behaviors were at a higher risk of patient delay (OR=2.13; 95% CI 1.04-4.36) in the unadjusted model. After adjustment for pediatric cancer patients’ socioeconomic characteristics (Model 1), a similar association was still observed (OR=2.07; 95% CI 1.01-4.26). After additional adjustment for caregivers’ socioeconomic characteristics (Model 2), the association did not change (OR=2.18; 95% CI 1.02-4.67). Finally, in the fully adjusted model (Model 3) controlling for patients’ socioeconomic characteristics, caregivers’ socioeconomic characteristics, cancer characteristics, and related health service characteristics, the OR was 2.21 (95% CI 1.03-4.75) for patients whose caregivers reported OHIS behaviors, compared with those who did not.

**Table 2:** Unadjusted and multivariate-adjusted odds ratios (ORs) and 95% CIs for delays in pediatric cancer according to the caregivers’ online health information–seeking (OHIS) behavior in China, 2021 (n=303).

Model	Caregiver’s OHIS behavior, OR (95% CI)	
	No (n=106, 35%)	Yes (n=197, 65%)
Unadjusted model	1.00 (reference)	2.13 (1.04-4.36)
Model 1^a^	1.00 (reference)	2.07 (1.01-4.26)
Model 2^b^	1.00 (reference)	2.18 (1.02-4.67)
Model 3^c^	1.00 (reference)	2.21 (1.03-4.75)

^a^Model 1: patient age and gender.

^b^Model 2: model 1 + caregiver age, gender, residency, educational status, marital status, employment status, private insurance status, household income per year, number of children.

^c^Model 3: model 2 + cancer type, distance to a primary care provider, distance toa a pediatric cancer specialist in Shandong province, number of health care providers visited before a diagnosis.

### Impact of OHIS Behaviors on the Cancer Care Pathway

Following the quantitative analysis, we interviewed 29 caregivers whose children had any one of the 3 types of delay: patient delay, diagnosis delay, or treatment delay. The interviews were coded into different categories, then into 7 subthemes, and finally into 2 themes using an iterative process. The majority (16/29, 55.2%) of the caregivers were mothers, and the pediatric patients had an average age of 6.14 (SD 3.36) years. Most (23/29, 79.3%) of the children were male, and over one-half (16/29, 55.2%) were diagnosed with pediatric solid tumors. More detailed information is presented in Table S2 in Multimedia Appendix 1.

### Symptom Appraisal by Caregivers Before the First Medical Contact

Caregivers did not notice bodily changes in their children until the symptoms had become so serious that they could not be ignored. Although most caregivers used search engines like Baidu or social media platforms like WeChat, TikTok, or other apps for their information sources, such information was devoid of qualified health science information. Most caregivers had no pediatric cancer awareness. Interestingly, we noticed that platforms for charitable donations, such as Shui-di-chou, offered opportunities to learn about diseases when individuals made donations.

Before my child's illness, I did not know anything about the early symptoms (of pediatric cancer), and I only heard about the names (of these diseases) when I donated to the Shui-Di-Chou from WeChat.

D24, mother of a 4-year-old boy diagnosed with acute lymphoma

Some participants indicated that they performed self-diagnoses after noticing the early symptoms of cancer based on online search results and their own life experience. From the caregiver’s perspective, early symptoms of some types of pediatric cancer were easily confused with signs of some other common pediatric diseases, such as flu or diarrhea. Furthermore, some participants indicated that they chose self-treatment after self-diagnosis without seeking medical help. The low quality of online health information might result in delayed medical contact and offer a false sense of control over the situation. Many participants delayed seeking medical care until their child’s symptoms were sufficiently serious enough or did not respond to self-treatment. Selected quotes are listed in Table 3.

The first symptom was vomiting, so I thought that the child had eaten too much and so I didn't care; later, the child felt tired after walking just a few steps, so I typed these symptoms into a search engine to find some solutions. I then bought some calcium supplements online. After two months, he had a fever, and then I chose to go to a tertiary hospital.

D12, father of a 3-year-old boy diagnosed with leukemia

**Table 3:** Quotes about symptom appraisal before the first medical contact by caregivers of patients with pediatric cancer in China, 2021 (n=29).

Caregivers’ symptom appraisal section	Quotes
Unnoticed until the symptoms become serious	D4: In March 2020, my child had leg pain, started to lose weight and had a yellow face in May, and had a blood test at the kindergarten after school started on June 1. They found high lymphocytes on June 15. D11: We did not take the little swelling in the right eye seriously. D23: Because of being busy at work, the child was taken by the grandmother. We didn’t notice the leg swelling in a timely manner. The lag time was around 1.5 months. D25: Because stomach pains are common in children and because children are too young to describe and recognize pain, I didn't pay attention to them until they vomited again and went to the hospital for diagnosis. D26: In winter, the child wore too many clothes to detect abnormalities in the stomach. The delay from finding symptoms to medication was about 20 days, causing the results of metastasis. D28: The symptoms started as calf pain, and no pelvic bone abnormality was detected, so there was a delay of 10 days.
Self-diagnosis	D10: At the end of the year of 2015, I found that my son had difficulty passing stool, I self-diagnosed it as constipation and did not seek medical help. D27: My child had foot pain, leg pain, and night sweats. We thought at first it was a weakness and growing bone pain, so we didn't pay attention to it. We went to the doctor when the child could not stand the pain. D12: The child had a yellowish color and did not go to the hospital, mistaking it for the normal color of a child's face. D15: In November 2017, when my child was 1 year old, she developed leg pain and could not walk. I thought she had fallen down.
Self -treatment	D12: The first symptom was vomiting, so I thought the child had accumulated food and didn't care. So, I went to the pharmacy and bought gastrointestinal tablets and Pepto-Bismol; later, the child felt tired after walking a few steps, so I bought some calcium supplements. After 2 months, he had a fever, and then I chose to go to a tertiary hospital. D21: My child was depressed, couldn't walk, was bloated, didn't sleep, and was losing weight, so I gave her stomach and appetite tablets. D22: My child had leg pain twice. I thought it was growing pains and gave him calcium tablets.

### Caregiver’s Acceptance of the HCP’s Diagnostic and Treatment Decision

Many caregivers were frustrated when faced with diagnosis uncertainty, even after several visits with health professionals from whom they sought online assistance. However, some caregivers felt helpless after reading the negative online examples of a bad prognosis and were confused with the amount of online information and the conflicting advice received. In contrast, some others found comfort through participation in online mutual support teams like the WeChat team on how to choose an oncologist and how to communicate with HCPs. Participants who reported online search strategies found that it became easier to communicate with HCPs, and they were more able to accept the diagnosis.

After the child's symptoms appeared, we interrogated remotely on the online health informatic platforms, but we were confused that we got different diagnoses from health care providers on different platforms.

D23, mother of a 4-year-old boy diagnosed with rhabdomyosarcoma

Most interviewees described a process to search for medical jargon, common causes, prognosis, and medical costs of pediatric cancer due to their unfamiliarity with pediatric cancer. Some of them even heard about pediatric cancer for the first time, because they thought that only older adults could be diagnosed with cancer. The main difficulties with accessing online health information reported by interviewees included the use of Internet tools, the selection of Internet information, and the identification of fake news on the Internet. Some participants searched for other similar patients’ successful experiences at the same hospitals in online mutual help teams like the WeChat group. These caregivers who reported OHIS behaviors tended not to challenge HCPs. Selected quotations are listed in Table S3 in Multimedia Appendix 1.

I'm not very good at (using) mobile phones, and I don't know how to find authoritative information online, so there is a lot of psychological pressure.

D12, father of a 3-year-old boy diagnosed with acute lymphoma

In the WeChat group, we hope to get general treatment ideas for the disease, the experience of patients, economic estimates, and other related information...Other patients’ successful experiences encouraged us a lot and helped us to accept the treatment plans.

D9, father of a 7-year-old boy diagnosed with kidney cancer

## Discussion

### Principal Findings

In this study, we found that caregivers with OHIS behaviors had a higher risk of patient delay among the different kinds of delays, compared with those without. Even after adjustment for patients’ characteristics, caregivers’ characteristics, cancer characteristics, and health service factors, the association did not change. Furthermore, our qualitative analyses showed that caregivers’ OHIS behaviors impacted the time intervals in the cancer care pathway by influencing caregivers’ symptom appraisal before the first medical contact and caregivers’ acceptance of the HCP’s diagnostic and treatment decision.

Few studies have explored the effect of caregivers’ OHIS behaviors on the development of a patient delay for pediatric cancer patients, and the findings have been inconsistent. A mixed methods study conducted in the United Kingdom suggested that caregivers’ OHIS did have an impact on patient delay [36]. In addition, a multisite qualitative study of survivors of gynecologic cancer [42] pointed out that caregivers’ OHIS behaviors may prolong a patient delay, which is consistent with what we found in this study. On the contrary, a study in Sweden suggested that caregivers’ OHIS behavior was a protective factor against delays before treatment. These inconsistent results may be due to differences in study sites and cancer types. Teh and colleagues [43] documented a discrepancy in the quality of websites between countries, with significantly more qualified resources available for patients with cancer in higher-income countries. For China, as a representative lower-income country, the quality of online health information was much lower than that in higher-income countries like Sweden. Therefore, the search results might be different for the same OHIS behaviors at different study sites. Furthermore, pediatric cancer is rare, and its symptoms are ambiguous; therefore, caregivers of patients with pediatric cancer might use inaccurate search words to seek help via the Internet. Papp and collogues [44] showed that early symptoms of childhood malignant diseases like pediatric cancer are not specific and that similar symptoms like fatigue, bone marrow failure, or pallor are often confusing. Chen and colleagues [45] also showed that incorrect initial diagnostic impressions are common among pediatric cancer caregivers. In this study, some participants were diagnosed with rhabdomyosarcoma or yolk sac tumor, which could be classified as rare diseases in China. Cancer types might affect the results of OHIS behaviors by using inaccurate search words due to the symptoms that are not obvious.

To prevent a patient delay, caregivers must interpret early symptoms in the context of cancer. The results of this study showed that Chinese caregivers of patients with pediatric cancer have limited knowledge about early symptoms of pediatric cancer, and most of them even heard about pediatric cancer for the first time when their children were diagnosed. These results are consistent with previous reports on Chinese caregivers’ low cancer awareness. The popularization of general online health science is growing fast in China. Xiong and colleagues [46] pointed out that health science popularization was the most searched-for information by Chinese online health information seekers. However, attention to the popularization of online health science for specific diseases like pediatric cancer is still needed. Ji and colleagues [47] compared the differences in OHIS behaviors between China and the United States, and the results showed that Chinese patients or caregivers were more likely to seek online health information after noticing symptoms, while US patients preferred to seek common medical knowledge online. In this study, we also found that the main sources of OHIS behaviors among Chinese caregivers of patients with pediatric cancer include search engines, WeChat, mobile apps, and new media. Zhang and colleagues [48] reached the same conclusion among the general population in China. In the near future, popularization of online health science for pediatric cancer through WeChat or search engines is needed to improve cancer awareness by Chinese caregivers of pediatric cancer patients.

A patient delay could occur when a patient with cancer or caregiver who presents with symptoms attributed to cancer does not seek medical help [49]. A review article published in Nature about delays in pediatric cancer showed that caregivers of patients with pediatric cancer may only seek medical attention once it is perceived to be necessary, creating increased lag time and delaying specialist contact and subsequent diagnosis [2]. Different caregivers might have different standards regarding the necessity of seeking professional medical help based on their previous perceived information about pediatric cancer. This study showed that caregivers of patients with pediatric cancer might face difficulties like the use of Internet tools, selection of Internet information, and identification of fake news on the Internet. Gao et al [50] described the difficulties of using Internet tools among older Chinese adults in the 21st century. Ulloa-Morales and colleagues [51] pointed out that online health information is characterized by incompleteness, not being useful, and not being scientifically supported. Except for the initial difficulties with searching online for information on pediatric cancer, lacking qualified online health information related to pediatric cancer was another factor contributing to the limited symptom appraisal in China. The online health information was filled with entertainment news, provided limited information on pediatric cancer, and was commercialized or fake [18]. It was hard to find qualified online health information for pediatric cancer from accredited experts or research institutions, and the sources need to be regulated to provide qualified online health information related to the early symptoms of pediatric cancer.

### Limitations

Some limitations should be acknowledged. First, recall bias might exist in some measures of this study. The time of first medical help for patients in our study was up to 9 years before study entry, and caregivers of patients with pediatric cancer were asked to recall events before the first medical help. For example, caregivers may not have been able to recall all the details of early symptoms noticed before their child’s first medical help and the characteristics of OHIS behaviors. However, it was not feasible for us to recruit participants prior to the first medical help. Second, although we demonstrated a higher risk of patient delay for patients with pediatric cancer whose caregivers reported OHIS behaviors, definitive causal relationships could not be inferred because our study was cross-sectional. However, the associations reported between the study variables were consistent with those in the existing literature. Third, this study just examined the association between caregivers’ OHIS behaviors and delays in pediatric cancer without deeply exploring the type, frequency, or intensity of caregivers’ OHIS behaviors due to limited funding. In future, studies of more detailed information regarding caregivers’ OHIS behavior could be used to verify this relationship. Fourth, there were various definitions of delays in pediatric cancer, and different time cutoffs for delays might lead to different results. In future studies, more types of delay definitions could be used to examine the relationship between caregivers’ OHIS behavior and delays in pediatric cancer. Fifth, all study participants were from Chinese hospitals with leading capabilities in pediatric blood tumors or solid tumors, which means that the generalizability of the findings might be limited for local medical capabilities of pediatric cancer and the cancer type. Furthermore, due to differences in cultural background and quality of online health information in the country, the extrapolation of the results needs to be further verified.

### Conclusions

Our study found that OHIS behaviors by caregivers are a risk factor for a patient delay in pediatric cancer, of the 3 different kinds of delays (patient delay, diagnosis delay, and treatment delay). These findings enhance our understanding of risk factors for patient delays and emphasize the need to address the risk of patient delay in patients with pediatric cancer whose caregivers demonstrate OHIS behaviors. Our government and society should make a concerted effort to regulate online health information and improve its quality. Specialized freemium consultations provided by HCPs via online health informatic platforms are needed to shorten the time for caregivers’ cancer symptom appraisal before the first medical contact. In addition, online mutual support for patient-patient interaction could bring hope and encouragement for patients with pediatric cancer and their caregivers.

## Appendix

Multimedia Appendix 1 Supplementary tables.

## Abbreviations

HCP: health care provider
OHIS: online health information–seeking

## Data Availability

The data sets used and analyzed in this study are available from the corresponding author upon reasonable request.
