# Supplementary material for: Association Between Online Health Information–Seeking Behaviors by Caregivers and Delays in Pediatric Cancer: Mixed Methods Study in China
Source: J Med Internet Res. 2023 Aug 16;25:e46953. doi: 10.2196/46953 (PMC10468701; doi:10.2196/46953)
Supplement: Multimedia Appendix 1 [file jmir_v25i1e46953_app1.docx]

| **Table S1.** Pearson Correlations among Caregiver’s Online Health Information Seeking (OHIS) and Various Types of Delays of Pediatric Cancer Patients in China, 2021 (N = 303). | | | | | |
| --- | --- | --- | --- | --- | --- |
| Variables | Caregiver’s OHIS | Delays of Pediatric Cancer | Patient Delay | Diagnosis Delay | Treatment Delay |
| Caregiver’s OHIS | 1 |  |  |  |  |
| Delays of Pediatric Cancer | 0.1083 | 1 |  |  |  |
| Patient Delay | 0.1210* | 0.4460*** | 1 |  |  |
| Diagnosis Delay | 0.0648 | 0.7737*** | 0.0433 | 1 |  |
| Treatment Delay | -0.0411 | 0.2943*** | -0.0316 | 0.1023 | 1 |
| *P<005; ***P<0.001 | | | | | |

|  |
| --- |

| **Table S2.** Summary Information about Caregivers included in the qualitative analysis in China, 2021 (N=29). | | | | | |
| --- | --- | --- | --- | --- | --- |
| **Code** | Role | Age of Children | Gender of Children | Date of diagnosis | Cancer type |
| D1 | Mother | 9 | Female | 2021.2 | Leukemia |
| D2 | Father | 12 | Male | 2020.12 | Leukemia |
| D3 | Father | 6 | Male | 2020.7 | Leukemia |
| D4 | Mother | 7 | Female | 2020.7 | Leukemia |
| D5 | Mother | 5 | Male | 2019.6 | Solid Tumors |
| D6 | Father | 13 | Male | 2019.5 | Solid Tumors |
| D7 | Mother | 4 | Male | 2019.3 | Solid Tumors |
| D8 | Father | 5 | Male | 2019.12 | Solid Tumors |
| D9 | Father | 7 | Male | 2020.7 | Solid Tumors |
| D10 | Mother | 7 | Male | 2016.5 | Solid Tumors |
| D11 | Father | 11 | Male | 2012.6 | Solid Tumors |
| D12 | Father | 3 | Male | 2019.8 | Leukemia |
| D13 | Father | 6 | Male | 2020.6 | Leukemia |
| D14 | Mother | 5 | Female | 2021.3 | Leukemia |
| D15 | Mother | 5 | Male | 2018.2 | Leukemia |
| D16 | Father | 6 | Female | 2020.10 | Leukemia |
| D17 | Mother | 13 | Male | 2020.9 | Leukemia |
| D18 | Father | 1 | Male | 2021.4 | Leukemia |
| D19 | Mother | 11 | Female | 2020.7 | Leukemia |
| D20 | Mother | 3 | Male | 2021.4 | Solid Tumors |
| D21 | Father | 3 | Male | 2020.8 | Solid Tumors |
| D22 | Father | 9 | Male | 2020.1 | Solid Tumors |
| D23 | Mother | 4 | Male | 2021.1 | Solid Tumors |
| D24 | Mother | 4 | Male | 2019.6 | Solid Tumors |
| D25 | Mother | 2 | Male | 2021.4 | Leukemia |
| D26 | Mother | 2 | Male | 2020.12 | Solid Tumors |
| D27 | Mother | 5 | Female | 2019.1 | Solid Tumors |
| D28 | Father | 7 | Male | 2019.1 | Solid Tumors |
| D29 | Mother | 3 | Male | 2019.10 | Solid Tumors |

| **Table S3.** Quotes from Caregivers of Pediatric Cancer Patients about Online Health Information Seeking (OHIS) before and after Symptom Detection in China, 2021 (N=29). | |
| --- | --- |
| Caregivers’ OHIS Section | Quotes |
| **Before Noticing the Early Symptoms** | ***D5:*** Although I have a medical background, I used to have less knowledge about pediatric oncology and only know about common blood tumors such as leukemia. |
|  | ***D24:*** Before my child's illness, I did not know any information about the early symptoms (of pediatric cancer), and I only heard about the names (of these diseases) when I donate to the Shui-Di-Chou from WeChat. |
|  | ***D29:*** Before the disease, I only knew about leukemia, but I had no idea about solid tumors. |
| **After Noticing the Early Symptoms** | ***D4 + D10+ D18+ D25 + D26 + D27 + D29:*** After learning some information, I will think about it and worry that my child will not be cured. |
|  | ***D6:*** There will be related science to make yourself more aware of the disease; there are also inquiries about the very seriousness of the disease that have an impact on the information. |
|  | ***D7:*** I think I can judge the online information about diseases and not be gullible which has a positive impact on the treatment and care of my child's diseases |
|  | ***D9:*** In the early stage, we hope that the public (parents) could learn basic knowledge about the initial symptoms of childhood cancer…later hope to get the general treatment ideas of the disease, the experience of patients, economic estimates, and other related information. |
|  | ***D12 + D14:*** I'm not very good at (using) mobile phones and I don't know how to find authoritative information online, so there is a lot of psychological pressure. |
|  | ***D21:*** It is believed that some of the results online are relatively optimistic and those actual recurrences of the blastoma are very frequent. |
|  | ***D22:*** I googled it myself, mainly to check the better medical institutions. |
|  | ***D23:*** On the internet,（after describing the symptoms）, some doctors took it seriously and some lightheartedly, causing misleading information, and the bad information caused psychological stress. |
| **Main Ways of OHIS** | ***D10 + D22 + D27 +D29:*** Baidu |
|  | ***D20:*** Baidu and WeChat. |
|  | ***D14+D21:*** Baidu and APPs. |
| **Hoping Ways for the Future Online Health Science Popularization** | ***D1+ D13+D14+D21+D22+D23:*** WeChat and Health Science Popularization of TikTok. |
|  | ***D2:*** WeChat. |
|  | ***D4:*** SMS, Phone Calls, APP, and WeChat. |
|  | ***D9 + D28:*** APP. |
|  | ***D27:*** Videos. |
| OHIS: Online Health Information Seeking | |
